# Interrater Reliability of mHealth App Rating Measures: Analysis of Top Depression and Smoking Cessation Apps

**DOI:** 10.2196/mhealth.5176

**Published:** 2016-02-10

**Authors:** Adam C Powell, John Torous, Steven Chan, Geoffrey Stephen Raynor, Erik Shwarts, Meghan Shanahan, Adam B Landman

**Affiliations:** ^1^ Payer+Provider Syndicate Boston, MA United States; ^2^ Harvard Longwood Psychiatry Residency Training Program Boston, MA United States; ^3^ Brigham and Women’s Hospital Department of Psychiatry Harvard Medical School Boston, MA United States; ^4^ University of California Davis Medical School Department of Psychiatry Sacramento, CA United States; ^5^ Brigham and Women’s Hospital Harvard Medical School Boston, MA United States

**Keywords:** mobile applications, mental health, evaluation studies, health apps, ratings

## Abstract

**Background:**

There are over 165,000 mHealth apps currently available to patients, but few have undergone an external quality review. Furthermore, no standardized review method exists, and little has been done to examine the consistency of the evaluation systems themselves.

**Objective:**

We sought to determine which measures for evaluating the quality of mHealth apps have the greatest interrater reliability.

**Methods:**

We identified 22 measures for evaluating the quality of apps from the literature. A panel of 6 reviewers reviewed the top 10 depression apps and 10 smoking cessation apps from the Apple iTunes App Store on these measures. Krippendorff’s alpha was calculated for each of the measures and reported by app category and in aggregate.

**Results:**

The measure for interactiveness and feedback was found to have the greatest overall interrater reliability (alpha=.69). Presence of password protection (alpha=.65), whether the app was uploaded by a health care agency (alpha=.63), the number of consumer ratings (alpha=.59), and several other measures had moderate interrater reliability (alphas>.5). There was the least agreement over whether apps had errors or performance issues (alpha=.15), stated advertising policies (alpha=.16), and were easy to use (alpha=.18). There were substantial differences in the interrater reliabilities of a number of measures when they were applied to depression versus smoking apps.

**Conclusions:**

We found wide variation in the interrater reliability of measures used to evaluate apps, and some measures are more robust across categories of apps than others. The measures with the highest degree of interrater reliability tended to be those that involved the least rater discretion. Clinical quality measures such as effectiveness, ease of use, and performance had relatively poor interrater reliability. Subsequent research is needed to determine consistent means for evaluating the performance of apps. Patients and clinicians should consider conducting their own assessments of apps, in conjunction with evaluating information from reviews.

## Introduction

Although there are over 165,000 mHealth apps currently available, few have undergone an external quality review [1,2]. Currently patients and doctors may find themselves turning to the Apple iTunes or Android Google Play app stores to identify which apps may be helpful and avoid those that may be ineffective or even harmful. The user ratings in these marketplaces are not designed to be a metric of medical appropriateness, safety, or efficacy of apps. Quality reviews conducted by trusted third-parties are important, as it is typically infeasible for clinicians and patients to evaluate the security, validity, and efficacy of apps. Third-party reviews have played an important role in highlighting the quality of enterprise software (eg, KLAS), consumer electronics (eg, Underwriters’ Laboratories), and even food (eg, Zagat) [3]. Health care providers and patients have a similar need for quality reviews of mHealth apps.

There are numerous challenges in rating apps. The availability of clinical data to guide app recommendations is poor. Medical research on apps is far behind. For example, while over 20 million American’s suffer from depression each year and there are over 1000 depression apps in consumer marketplaces [4], a recent review found only 10 published studies on depression apps [5]. The United States Food and Drug Administration does not offer guidance either, noting it does not plan to regulate many apps that are of low risk [6]. The efforts of professional and regulatory bodies to curate apps have also been disappointing, with the recent news of the shutdown of the British National Health Service’s Health App Library [7] after a study revealed that many apps accredited actually transmitted medical data in an unsecure manner and that several lacked privacy policies [8], among other concerns. Finally, any app rating system would have to be continually updated, as apps themselves are often upgrading and changing.

Despite the substantial challenges in rating apps, there have been important efforts to begin to understand how to best approach the problem. Several methodologies have been proposed for evaluating the quality of apps [9-11], although no standardized method exists, and little has been done to examine the consistency of the evaluation systems themselves.

One methodology of note that was introduced after the design of this study is the Mobile App Rating Scale (MARS), a 23-item scale that demonstrates strong internal consistency and interrater reliability in a research study involving 2 expert raters [12]. However, like many scales, its validity is still uncertain and it has not been widely adopted yet. Furthermore, there have been both generalized frameworks [11] and specialty-specific efforts [13-15] that use inconsistent measures to evaluate quality. However, it is unclear how any of these methodologies and scales hold up in real-world clinical practice and when used by non-expert raters. Given the rapid growth in the number of mHealth apps and the constant updating of existing apps, it makes sense that clinicians will need to utilize and apply app ratings [2].

The need for quality measures of mHealth apps will become increasingly important as more patients ask physicians for app recommendations. The lack of standardized quality measures is concerning, as app use carries risk and can lead to adverse outcomes for both patients and clinicians. Poorly designed apps may offer ineffective care or even cause harm [16]. Clinicians may place themselves at legal, professional, and ethical risk if digital technologies are recommended inappropriately [17]. Furthermore, if apps are eventually to be prescribed by providers and reimbursed by payers, there must be a robust way to evaluate the safety and effectiveness of apps [18]. Standardized measures with high interrater reliability are needed to evaluate mHealth apps. Nonetheless, without standardized measures for evaluating app outcomes, clinicians have difficulty comparing the quality of apps when making recommendations. When measures are available, quality measures with low interrater reliability are problematic, as ratings produced are not necessarily reflective of what would be experienced by other raters or users.

In order to foster the development of standardized app quality measures, we seek to evaluate the interrater reliability of existing measures [19] and provide direction on which should be incorporated in standardized app evaluation systems. In addition, we seek to determine whether the interrater reliability of the measures is consistent across multiple types of apps, and which of these measures may be the best to incorporate into app evaluation systems and tools. As mHealth apps are used for a wide range of purposes, it is important to understand which quality measures consistently perform with high interrater reliability, regardless of the nature of the apps being evaluated.

## Methods

### Selection of Apps

To evaluate whether the interrater reliability of quality measures is consistent across multiple types of apps, we evaluated the top 10 search results displayed by the US Apple iTunes App Store for iOS (iPhone, iPod Touch, and iPad) returned by queries for “depression” and “smoking” in March 2015 (see Multimedia Appendix 1). While rankings are in constant flux as apps are updated and rated by app store users, the apps selected reflect those most visible to users seeking assistance at the time of selection. This was done to increase the realism of the sample, as users without pre-existing knowledge of which apps to seek are likely to pick those they see first.

Depression and smoking cessation (hereafter referred to as “smoking”) categories were selected because they are common issues, cause significant comorbidity, and have been the targets of many early mobile phone interventions that are directly marketed to patients [20-22]. In 2013, over 15 million US adults suffered from depression (6.7% of the population) [23], and over 42 million Americans smoked cigarettes (nearly 18% of the population) [24]. Also, a recent study suggested that when considering apps by treatment type, mental health and behavioral disorders are the largest sector—more numerous than both cardiac and cancer-focused apps [25]. The clinical evidence supporting apps in these categories has the potential to be more robust than supporting apps managing other indications [26].

### Selection of App Quality Measures

We reviewed existing mHealth app ratings sites and the literature to identify app evaluation measures. At this time, there is no gold standard for rating apps and no central repository of app ratings. While the list of measures evaluated may not be definitive, it is varied and reflective of the practice of a number of organizations. The Anxiety and Depression Association of America (ADAA) and PsyberGuide were two websites that were used as sources of measures. The ADAA rates apps on ease of use, perceived effectiveness, personalization, interactiveness/feedback, and research evidence on a 5-point scale [27]. PsyberGuide evaluates the basis of the research behind the app, the source of the funding for the research, the specificity of the proposed intervention, the number of consumer ratings, whether a product advisory board with clinical thought leadership exists, and whether the app has been revised within the past 12 months [28].

Although the ADAA and PsyberGuide measures were the best characterized, we also used measures cited in the literature, including whether an app has password protection and import/export capabilities [29], whether an app is uploaded by a health care agency versus a non-health care agency [30], whether the developer is contactable and the advertising policy is clearly stated [31], whether there is a lack of errors or hang-ups and continuous access to the data [32], whether an app discloses potential risks, and whether it offers technical support or help [33].

mHealth apps must be safe, accurate, effective, secure, and protect privacy to be used by patients, recommended by health care professionals, and eventually reimbursed [2]. While it is difficult to assess the safety or accuracy of an app without an in-depth review, it is possible to rapidly determine whether an app has security measures such as password protection and encryption, and privacy measures such as an explicit privacy policy. Likewise, it can be difficult to assess the effectiveness of an app. We included three measures of effectiveness: perceived effectiveness, research evidence base for an app, and whether or not the app claimed that the effectiveness was tested. Evaluating perceived effectiveness required the reviewers to subjectively evaluate the app against its stated objective, whereas the evidence base and statement of effectiveness made by the app were evaluated based on discrete findings. Table 1 summarizes all of the app quality measures included in this study and presents the terms of each measure exactly as defined for reviewers.

### App Review Process

Each app was rated by up to 6 reviewers. Reviewers were an interdisciplinary group including clinicians, technology experts, and researchers; all are study co-authors. Reviewers were instructed to rate all 20 apps (Multimedia Appendix 1) on all 22 measures (see Table 1). Each reviewer reviewed a minimum of 17 of the 20 apps on each of the 22 measures.

Reviews were conducted between March and May 2015. All apps were reviewed running on iPhones. Reviewers did not discuss their reviews with each other to ensure that each rating was independent. For each of the apps, the reviewers were provided a copy of Table 1, with space for their ratings. Reviewers were not provided with any additional training in the review methodology in order to best approximate what would occur if the reviewers read about the metrics independently. No human subjects were used in this study. Results are reported in aggregate only, not at the app level.

Reviewers were asked to download the apps to their personal iPhones and then to assign values to each of the measures using only information provided within the app itself and in the iTunes App Store for iOS. As reviewers used their personal iPhones, it is likely that multiple hardware and operating system configurations were used during the review process. Although reviewers were asked to spend several minutes examining each app, they were not asked to use the apps in a realistic manner. Realistic use would not have been feasible, as the reviewers were selected for their expertise in mHealth, rather than for their history of depression or smoking. This short duration of use simulates what is likely to occur when apps are evaluated by experts without a personal need for the apps in question.

After reviews were completed, the reviewers were sent screenshots from each of the apps and asked to verify that the apps they reviewed were the same as the ones shown. One reviewer was unable to review one smoking app and misidentified a second smoking app and one depression app. In the cases where apps were misidentified, they were treated as missing. Each measure was applied up to 120 times (20 apps rated by 6 reviewers). However, due to app identification issues and occasional cases of reviewer uncertainty about the proper rating to apply, each measure was only applied between 109 and 112 times, yielding data completeness between 91% and 93%, depending on the measure (see Table 2).

**Table 1:** mHealth app quality measures evaluated.

Measure	Source	Range	Definitions
Ease of use	ADAA	1-5	5=very easy; 1=very difficult
Effectiveness (Perceived)	ADAA	1-5	5=highly likely; 1=highly unlikely
Personalization	ADAA	1-5	5=complete ability; 1=no ability
Interactiveness/Feedback	ADAA	1-5	5=very interactive, helpful feedback; 1=not interactive, no feedback
Basis of research	PsyberGuide (& ADAA^a^)	0-3 (and 1-5^a^)	3=data from at least one randomized controlled trial; 2=data from at least one non-randomized non-controlled trial; 1=data from an open study; 0=no data provided
Source of funding for research	PsyberGuide	0-2	2=research supported exclusively by government agency or non-profit organizations; 1=research supported in full or part by for-profit organizations; 0=no data provided
Specificity of intervention	PsyberGuide	1-3	3=the application is designed to improve a specific condition or symptom; 2=the application is designed to help with non-specific items such as “mood” or “brain fitness”; 1=the application is designed to track and monitor items such as symptom severity or medication; 0= no data provided
Number of consumer ratings	PsyberGuide	1-3	3=ratings exist from >50 users; 2=ratings exist from 25-50 users; 1=fewer than 25 user ratings
Product advisory support	PsyberGuide	0-1	1=yes; 0=no
Software support	PsyberGuide	0-1	1=yes; 0=no
Password protection	Kharrazi et al (2012)	0-1	1=yes; 0=no
Import/export capabilities	Kharrazi et al (2012)	0-1	1=yes; 0=no
Uploaded by health care agency	Pandey et al (2012)	0-1	1=yes; 0=no
Encryption	Powell et al (2014)	0-1	1=yes; 0=no
Explicit privacy policy	Powell et al (2014)	0-1	1=yes; 0=no
Effectiveness tested (claimed by app)	Powell et al (2014)	0-1	1=yes; 0=no
Developer contactable	Lewis	0-1	1=yes; 0=no
Advertising policy stated	Lewis	0-1	1=yes; 0=no
Errors and performance issues	Martinez-Perez et al (2013)	0-1	1=yes; 0=no
Continuous availability of data	Martinez-Perez et al (2013)	0-1	1=yes; 0=no
Discloses potential risks	Ferrero-Álvarez- Rementería et al (2013)	0-1	1=yes; 0=no
Offers technical support or help	Ferrero-Álvarez- Rementería et al (2013)	0-1	1=yes; 0=no

^a^1=no research evidence; 5=ample research evidence; ADAA scale not used.

### Data Analysis

Interrater reliability for each app quality measure was evaluated for both the depression and smoking categories separately, as well as for the two categories combined. Krippendorff’s alpha was used to assess interrater reliability, as it allows for ordinal ratings to be assigned, can be used with an unlimited number of reviewers, is robust to missing data, and is superior to Cohen’s kappa [34-37]. An alpha ˃.667 was used to indicate agreement [35]. A negative alpha indicates less agreement than would be expected by chance and indicates that there may have been inconsistencies in how measures were applied. Krippendorff’s alpha was calculated using the krippalpha module for Stata [38].

## Results

Table 2 summarizes the interrater reliability of app quality measures overall and by application type, that is, depression or smoking. The level of data completeness for each measure is additionally reported. When considered in aggregate, only the measure for interactiveness and feedback reached our threshold for agreement. However, a number of other measures came close, with alphas ˃.5: presence of password protection, whether the app was uploaded by a health care agency, number of consumer ratings, whether the app had an explicit privacy policy, whether the app had encryption, whether the app was based on research, and whether the app had product advisory support. There was the least agreement over whether apps had errors or performance issues, stated advertising policies, were easy to use, made claims about effectiveness being tested, and made data continuously available.

When ratings for depression apps were evaluated independently, both interactiveness and feedback measure and the password protection measure reached our threshold for agreement. Meanwhile, when ratings for smoking apps were evaluated independently, there was perfect agreement (alpha=1) on whether the apps were uploaded by health care agencies and whether the apps were encrypted. There were also differences in app quality measure reliability between the depression and smoking apps. The difference in alpha when applied to depression versus smoking apps was greater than .4 for the following measures: encryption, import/export capabilities, and specificity of intervention.

**Table 2:** Interrater reliability of depression and smoking apps by measure.

Measure	Interrater reliability (Krippendorff’s alpha)			Completeness, %
	Aggregate	Depression	Smoking	
Interactiveness/Feedback	0.69	0.69	0.67	93
Password protection	0.65	0.75	0.37	93
Uploaded by health care agency	0.63	0.60	1.00	93
Number of consumer ratings	0.59	0.74	0.42	93
Explicit privacy policy	0.55	0.73	0.38	93
Encryption	0.54	0.51	1.00	92
Basis of research	0.53	0.55	0.44	93
Product advisory support	0.52	0.55	0.44	93
Offers technical support or help	0.45	0.50	0.35	93
Software support	0.44	0.42	0.44	93
Import/export capabilities	0.42	0.47	0.04	93
Developer contactable	0.42	0.38	0.36	93
Personalization	0.42	0.38	0.49	93
Specificity of intervention	0.36	0.33	-0.14	91
Source of funding for research	0.36	0.22	0.59	92
Discloses potential risks	0.31	0.23	0.00	93
Effectiveness (Perceived)	0.30	0.43	0.12	93
Continuous availability of data	0.27	0.22	0.09	93
Effectiveness tested (claimed by app)	0.21	0.11	0.34	93
Ease of use	0.18	0.09	0.23	93
Advertising policy stated	0.16	-0.04	0.20	93
Errors and performance issues	0.15	0.28	0.03	93

## Discussion

### Principal Findings

Overall, we found only a few measures with high interrater reliability; most of the app measures had poor interrater reliability. The measures with the highest degree of interrater reliability tended to involve the least rater discretion. For instance, the presence of encryption and whether or not the app was uploaded by a health care agency can be assessed largely on a factual basis, while the ease of use measure is subject to interpretation. Surprisingly, the presence of interactiveness/feedback, the measure with the greatest degree of interrater reliability overall, was a subjective measure. This suggests that raters were able to come to directional agreement about interactiveness/feedback. While the measure was subjective, it may have led to more consistent ratings than many of the objective measures, as it could be assessed by examining an app holistically rather than by correctly identifying a single feature. While the presence or absence of a feature can be objectively determined, it can also more easily be missed by a reviewer performing a rapid review.

For mHealth apps to be successfully used in clinical practice, they need to be safe and effective. Reviewers had moderate agreement on security/privacy measures, including the presence of password protection, explicit privacy policy, and encryption. Perceived effectiveness had low interrater reliability suggesting that even finding agreement on which apps may actually offer effectiveness is challenging. Effectiveness tested as claimed by the app also had low interrater reliability. The basis of research behind an app had better interrater reliability and may be a more reliable indicator of effectiveness than perceived effectiveness. Due to the current limited data on the efficacy of most apps, relying on the research base alone is difficult at this time. Given the increasing shift to value-based care with rewards for delivering high-quality care based on evidence-based principles, subsequent research may examine ways of more consistently evaluating the performance of apps on these useful dimensions. Automated outcomes reporting may be a means of gauging effectiveness, and automated crash reporting may be a means of gauging performance [3].

Beyond effectiveness, other clinically important measures, such as ease of use and performance issues, also had relatively poor interrater reliability. Reviewers rated perceived effectiveness and ease of use with 5-point Likert scales in this study. More robust measures are needed for these important measures. The validated System Usability Scale [39] could be considered to measure ease of use.

We also found differences in the performance of measures between the smoking and depression apps. Depression apps had much higher interrater reliability on the presence of password protection than did smoking apps. Several of the smoking apps had passwords associated with the social network functionality, but not the rest of the app, which may have been a source of ambiguity for reviewers. The measures on whether an app was uploaded by a health care agency and contained encryption had perfect agreement for smoking apps, but not for depression apps. Therefore, there may be fundamental differences between categories of apps that impact the interrater reliability of measures. As a result of this heterogeneity, it may be necessary to validate the interrater reliability of measures separately for each category of apps.

While the challenge of rating mHealth apps may be new, issues related to rating scales are not new to health care. Measuring quality of care of hospitals and health care systems is challenging, and there is also at times little consensus between various metrics [40]. Perhaps organizations seeking to rate apps can learn from the quality improvement and safety literature to understand how to best implement effective rating scales.

Overall, the results suggest a need for great caution when assigning and interpreting app ratings (see Table 3). Organizations rating apps should create robust measures with clearly defined criteria to ensure consistency. They should then test the interrater reliability and validity of their measures and utilize only ones with high levels of agreement. The reliability of reviews can further be improved by carefully training reviewers on how to consistently apply the measures. Consumers of reviews—both patients and clinicians—should interpret reviews cautiously, especially if they rely on measures that have not been shown to be reliable and valid. Clinicians may wish to use reviews as a mechanism for selecting potentially beneficial apps and then carefully test apps before recommending them to patients to ensure that the reviewers’ definitions of quality are consistent with their own. There may likewise be value in discussing apps with colleagues before recommending them, as it appears that clinicians may not identify quality issues consistently. Even in this era of digital health, clinician judgment may still be the best available tool for evaluating apps. Table 3 summarizes lessons learned from our app review.

**Table 3:** Key lessons learned for clinicians, patients, and app reviewers.

For clinicians	For patients	For app reviewers
Interpret mHealth app reviews cautiously, especially if measures have not been validated	Interpret mHealth app reviews cautiously	Use previously validated measures with high interrater reliability, if available
Consider reviewing apps personally before recommending apps to patients	Consult with your health care provider or another trusted source	Train reviewers on the measures using standardized specifications
Consider discussing apps with colleagues		Involve patients or reviewers with the condition of interest in the reviews
Use clinical judgment as a tool for evaluating apps		Record the name and version of the app being reviewed, as well as the date of the review

### Limitations

While this study provides some initial conclusions about interrater reliability, it may have limited generalizability and does not address measure validity. Only 20 apps in two categories were evaluated, and some differences were found in the interrater reliability of the measures across categories. We included only Apple iOS apps for iPhone devices; Android-only apps were not considered. Furthermore, the apps selected may not be representative of their respective categories.

The app review methods used may have contributed to low measure interrater reliability. The measures of review completeness suggest that reviewers sometimes had issues determining the appropriate ratings for measures or finding the right apps. Future researchers and physicians prescribing apps should consider providing direct links to apps to ensure that there is no confusion. It is easy to imagine situations where physicians recommend apps to patients by name, and then patients are unable to properly identify apps as other available apps have similar titles. Likewise, detailed measure definitions and additional reviewer training may help future reviewers provide more complete ratings with confidence. Nonetheless, each measure was applied 109 or more times, out of a potential total of 120 applications, indicating that in the majority of cases, reviewers were able to properly identify the app and apply a rating for the measure. Further, there may be idiosyncrasies in the rating behavior of the 6 reviewers who participated.

Of note, we did not include any patient ratings in our review process. In the future, it would be interesting to compare the ratings of patients versus expert reviewers. It is possible that patients with depression or tobacco use disorder may have been able to identify salient app features that our reviewers missed. Further research is needed in this important area.

Last, reviews were performed over a 3-month period between March and May 2015, and it is possible that the apps themselves evolved through updates during the period in a way that impacted the reviews. While this issue impacts interrater reliability, it also impacts the usefulness of reviews, as patients and clinicians may sometimes have to rely on reviews that were conducted on prior versions of apps.

### Conclusions

Our study suggests that some measures have greater interrater reliability than others and that the relative interrater reliability of measures is not robust across categories of mHealth apps. Unfortunately, the research also suggests that some of the most clinically useful measures—effectiveness and ease of use—have relatively poor interrater reliability. Organizations seeking to rate apps should consider the interrater reliability of the metrics they are utilizing and select metrics with lower levels of ambiguity. Clinician judgment thus remains critical in evaluating and understanding the clinical role of mobile phone apps.

## Appendix

Multimedia Appendix 1 mHealth apps.

## Abbreviations

ADAA: Anxiety and Depression Association of America
