# Supplementary material for: Interrater Reliability of mHealth App Rating Measures: Analysis of Top Depression and Smoking Cessation Apps
Source: JMIR Mhealth Uhealth. 2016 Feb 10;4(1):e15. doi: 10.2196/mhealth.5176 (PMC4766362; doi:10.2196/mhealth.5176)
Supplement: Multimedia Appendix 1 [file mhealth_v4i1e15_app1.pdf]

Multimedia Appendix 1. Table A1. mHealth apps reviewed.

Smoking	Depression
"Quit It Lite - stop smoking today" by digitalsirup GmbH	"Mental Illness: Facts on Anxiety, Depression, Bipolar Disorder, ADHD, PTSD, OCD Test, Screening, Treatment and Self Help Management Free!" by Michael Quach
"LIVESTRONG MyQuit Coach - Dare to Quit Smoking" by Demand Media, Inc.	"STAT Depression Screening PHQ-9" by Austin Physician Productivity, LLC
"Free Butt Out - Quit Smoking Forever" ("FREE Stop Smoking" at the time of review) by Ellisapps Inc.	"7 Cups of Tea – Free Anxiety Relief, Depression Help, Therapy & Counseling" by 7 Cups of Tea
"Smoke Free - Quit smoking now and stop for good" by David Crane	"depressioncheck" by M-3 Information, LLC
"Quit Pro: stop smoking now" by Bitsmedia Pte Ltd	"Surviving Depression" by Pauline Books and Media
"My Last Cigarette - Stop Smoking Stay Quit" by Mastersoft Ltd	"iPharmacy Drug Guide & PubMed Direct" by SigmaPhone LLC
"Quit smoking - QuitNow!" by Fewlaps, S.C	"Hamilton Depression Scale Lite" by openAirWare LLC
"Quit Smoking Hypnosis - FREE Guided Meditation and the Best Hypnotherapy Program to Help Stop Smoking Cigarettes Now" by Surf City Apps LLC	"Doctor On Demand: Live MD & Therapy Visits, w/ Board-Certified Physicians and Psychologists" by Doctor On Demand, Inc
"Quit Smoking Helper" by Tae-han Kim	"Stress Check by Azumio – Lite" by Azumio Inc.
"Stop Smoking!" by On Beat Limited	"T2 Mood Tracker" by "The National Center for Telehealth and Technology"
